# Factors impacting sustainability of community health worker programming in rural Uganda: a qualitative study

**DOI:** 10.4314/ahs.v22i2.76

**Published:** 2022-06

**Authors:** Scholastic Ashaba, Manasseh Tumuhimbise, Esther Beebwa, Francis Oriokot, Jennifer L Brenner, Jerome Kabakyenga

**Affiliations:** 1 Department of Psychiatry, Mbarara University of Science and Technology; 2 Department of Accounting and Finance, Mbarara University of Science and Technology; 3 Department of Nursing, Mbarara University of Science and Technology; 4 Department of Pediatrics and Child Health Mbarara Regional Referral Hospital; 5 Departments of Pediatrics and Community Health Sciences, Cumming School of Medicine, University of Calgary, Calgary, Alberta, Canada; 6 Institute of Maternal Newborn and Child Health, Mbarara University of Science and Technology

**Keywords:** CHWs programs, sustainability, maternal newborn and child health, Uganda

## Abstract

**Background:**

Despite significant global progress towards decreased child mortality in past decades, maternal and child mortality continues to be high, especially in sub Saharan Africa. Most of these deaths are preventable with known interventions. Community health workers (CHWs) are well-positioned to promote these life-saving interventions; however, sustaining CHW programs remains a challenge.

**Methods:**

A sustainability-focused qualitative evaluation, was done between July and August 2018 in 2 rural districts in southwest Uganda. Using semi-structured interview tools, we conducted 6 Focus Group discussions (FGDs) with CHWs and 17 in-depth interviews (IDIs) with various district stakeholders to gain insights into factors affecting sustainability of a district-wide maternal, newborn and child health (MNCH)-oriented CHW intervention. Data was managed using NVivo software (version 12) with themes using thematic analysis.

**Results:**

Identified factors impacting CHW program sustainability included ‘health system effectiveness’ (availability of supplies, medicines and services and availability of facility health providers), CHW program-related factors' (CHW selection and training, CHW recognition), ‘community attitudes and beliefs’ and ‘stakeholder engagement’.

**Conclusion:**

To sustain CHW programs in rural Uganda and globally, planners, policymakers and funders should maximize community engagement in establishing CHW networks and strengthen accountability, supply chains and linkages with communities and health facilities

## Background

Despite significant global progress towards decreased child mortality over the past decades, more than 5 million children die each year before reaching their fifth birthday^1^. Roughly half of these deaths occur in sub-Saharan Africa (SSA)^2^,^3^. Global estimates of women dying during pregnancy and childbirth in 2017 were 295,000; approximately two-thirds of deaths were in Sub-Saharan Africa (SSA)^4^,^5^. The vast majority (94%) of maternal and child deaths are preventable with known interventions^3^. A combination of service provision and community-based strategies are needed.

Globally, Community Health workers (CHWs) who promote healthy household practices and care seeking, have proven effective in improving health outcome indicators including maternal and child mortality^6^–^8^. This is true in SSA and in Uganda 9–11. A huge global investment in initiating CHW programs has resulted in CHWs now playing a critical role in health promotion and programing in many low income countries and settings^12^. CHW programs have been established in many SSA countries; community and national-level scale up is increasing. The Government of Uganda continues to identify maternal, newborn and child health (MNCH) programming as a priority^13^ and has national policies encouraging community-based approaches to health promotion^14^–^16^. Specifically, the Village Health Team approach^17^, initiated by the Ministry of Health in 2001, involves mobilization of networks of volunteer CHWs countrywide (known as Village Health Teams (VHTs). Selected from within villages, these CHWs are intended to strengthen ‘demand-side’ care-seeking and home practices and bringing facilities and communities closer together to garner improved health^12^,^14^,^18^.

CHWs have been instrumental and improved MNCH outcomes have been documented including increased antenatal care visits, facility-based deliveries and newborn health practices^19^,^20^. Previous studies in southwestern Uganda by researchers from our institution have documented improved child health practices, reduced malnutrition and reduced child deaths in southwestern Ugandan communities following introduction of volunteer CHWs^21^. Many studies have documented the success of district and national MNCH packages in Africa^22^ and Uganda that include antenatal care, post-natal care, breastfeeding, immunization, and skilled birth among others^23^,^24^. The role of CHWs in increasing awareness about birth preparedness and an increase of male involvement in maternal and newborn health has also been documented^25^.

However, in Uganda and globally, continuing to maintain CHW programs, especially at scale, remains a challenge. Little is known about if and how to sustain such networks. The majority of CHW effectiveness studies report outcomes during the project intervention period with limited documentation of sustainability post-intervention^26^,^27^. Globally, studies to understand barriers and enablers to CHW program scale up and sustainability are few28. Of studies published in SSA, most relate to HIV/AIDS programming^28^,^29^. With growing global pressure and efforts to scale up MNCH-focused CHW programs, there is a critical need to understand if and how sustainable different program types are in different contexts before huge scale up investments are made. In the longer term, CHW programs can only have medium and long-term impacts on maternal and child outcomes if sustained beyond initial implementation periods.

In community health programming, ‘sustainability’ refers to continued use of intervention components and activities aiming to achieve desirable health outcomes within the population of interest after the implementation phase has been completed^30^,^31^. A successful intervention involves continuity of relevant activities and resources congruent with the original objectives^32^. Common factors previously documented to promote sustainability include ‘management and supervision’, ‘use of existing community structures’ and ‘integration within the existing health system’^29^,^33^–^38^

Between 2012 and 2014, a comprehensive MNCH-focused CHW intervention was scaled up in two rural Ugandan districts. The initiative, undertaken by a university-district partnership (called Healthy Child Uganda) with Global Affairs Canada funding, took a district-wide approach to programming, combining service (health facility) and demand side (community) activities. By project end, key MNCH indicators improved including care-seeking for antenatal, delivery and postnatal services with reduced prevalence of pneumonia, diarrhea and underweight status^21^,^39^. Importantly, a network of over 2000 MNCH-focused volunteer CHWs was established and still active. Recent follow up has documented over 80% retention of these volunteers post initial training (unpublished) which is similar to other experiences reported elsewhere in Uganda^40^. However, factors associated with sustainability of these CHW cohorts have not been explored. This study aimed at exploring, in depth, factors associated with sustainability of CHW networks using qualitative methodology.

## Method

### Study Setting

Between 2012 and 2014, the Healthy Child Uganda partnership together with two rural Ugandan districts implemented a comprehensive MNCH package. Bushenyi and Rubirizi districts, located in a hilly region in southwest Uganda, have a combined population of approximately 350,000 people^41^. Most families are subsistence farmers, living in extreme poverty; communities are quite scattered. Poor roads and distance often challenge transport to health facilities^42^. Rubirizi district has 18 health facilities (1 Health centre IV, 6 health centre IIIs, 11 Health centre IIs). Bushenyi district has 39 health facilities including two private hospitals (2 Health centre IVs, 12 Health centre IIIs and 22 Health centre IIs)^41^.

### CHW Intervention

An MNCH intervention was implemented district-wide in both districts. Development of CHW networks were part of a larger MNCH capacity-building package. The package, which incorporated national MNCH and CHW guidelines and built on past community-based programming experiences, came to be known as ‘MamaToto’ (mother-child in Swahili). MamaToto activities occurred at three levels: (1) district (i.e health system strengthening such as data collection, transport and planning); (2) health facility (i.e clinical, management, governance, infrastructure); and (3) Community (i.e CHWs). Initial sensitization meetings in each parish involved orientation of community members and local leaders to the national CHW program and described expected CHW roles and conditions. Communities identified and selected CHWs in each village according to government recruitment guidelines through a process and criteria developed by each community. Common criteria included being a parent, active community involvement, demonstrated voluntary spirit, and being a trusted and respected community member.

In total, 2626 CHWs (69% female) received initial (5 day) training in MNCH health promotion and community development according to the government curriculum. One ‘refresher’ workshop lasting 3 days was provided. The training was based on the national curriculum and followed the government manual and guidelines^16^,^43^, complemented by participatory methods found to be successful in past community programming by Healthy Child Uganda (HCU). The five-day curriculum and two-day refreshers were designed to build health promotion skills and knowledge related to MNCH, including nutrition, birth preparedness and newborn care. Both trainings were conducted by district health workers who had undertaken training of trainers' workshops. CHWs were organized into teams which included members representing a number of villages. Teams were assigned a health worker from their designated facility for supervision and monthly report submission. CHW services to their community are voluntary. During the intervention, the only incentives provided were T-shirts, umbrellas, soap and a travel allowance (5,000Sh∼$2CAD) for training days only. Five years' post-intervention, this sustainability-focused qualitative evaluation was conducted (July/August 2018).

### Sampling and study participants

Purposeful selection was used to identify study participants from MamaToto intervention districts. The number of focus group discussions (FGDs) was determined by the available CHWs in sub-counties that were purposively selected. From a total 24 intervention sub-counties (geopolitical unit), 3 were purposively selected from each district based on the local leader input about perceived level of activity of CHWs in relation to fulfilling their responsibilities (i.e community mobilization for available services, community meetings frequency, and completion rates of monthly reports submitted to the health facility. Following this criteria, we selected sub county from each district with high performing CHWs, with average performing CHWs and lower performance CHWs. From each of the selected sub counties CHWs were randomly selected to participate in the FGDs each comprising of 10–12 participants. Three FGDs were conducted in each district).

In-depth interview (IDIs) participants were chosen based on their potential to contribute meaningfully to the topic areas (i.e sustainability) from a variety of perspectives. IDI participants included key district decision-makers, implementation field team members, and community leaders familiar with the MamaToto intervention. We purposively selected health unit management committee chairpersons, health facility in charges and health assistants that were directly involved in supervising the CHWs and who were working in the districts at the time of intervention implementation. We also interviewed community development officers who were working in the district at the time of project implementation and local leaders who were involved in CHWs work in different villages in the selected sub counties. The number of FGDs and IDIs was guided by previous research that thematic saturation can be achieved with 12–16 in-depth interviews^44^,^45^.

### Data Collection

FGDs and IDIs were designed to collect insights into post-intervention sustainability factors. Semi-structured interview guides were developed by research team members. Trained interviewers, fluent in English and local language (Runyankore), facilitated semi-structured FGDs and IDIs. Interviews were digitally recorded then translated and transcribed directly into English.

### Ethical considerations

Study approval was granted by the Research Ethics Committee of Mbarara University of Science and Technology (#04/06-17), Uganda National Council for Science and Technology (#SS4386), and the University of Calgary Conjoint Ethics Board (REB17-1741). Written informed consent was obtained from all study participants in their language of choice.

### Data Analysis

Data was managed using NVivo software (version 12, QSR International, Burlington Mass.). Study team members (SA, MT, JK, EB, and FO) conducted analysis. Thematic content analysis was used to analyse the data using an iterative process to identify themes relevant to sustainability of CHW programmes. Each analyst conducted an initial review of 5 pre-selected transcripts following which they highlighted emerging themes. Analysts then compared findings, discussed and harmonized differences and generated a code book that comprised of major themes. Analysts used these to code the remaining interviews independently. During coding, new thematic categories, patterns and linkages were explored. Triangulation was used to merge generated data from different interviews and group types (i.e CHWs and district stakeholders).

## Results

### Participant Characteristics

FGDs involved a total of 62 CHWs (32 males, 30 females), divided into 6 groups with 10–12 participants each. IDIs involved 17 district stakeholders including health providers (4 health assistants, 3 clinicians), community development officers (4), and community leaders (3 elected community leaders, 3 Health Unit Management Committee chairpersons). Mean participant age was 42.6 years (SD+/-10.5). More than half (51.6%) were male; 56% were married; the majority (61%) had attained secondary education.

### Factors impacting sustainability

#### Factors related to CHW sustainability were grouped into four thematic categories.

##### 1) Health system factors

Participants reported key health system factors affecting CHW programming sustainability. Staffing and supply shortages were reported to negatively affect CHW activity level and potential barriers to their sustainability.

*Availability of Supplies, Medicines and Services:* CHWs described feeling dispirited when they perceived doing a good job creating demand, yet clients they mobilized to seek care at facilities found medical supplies and medicines lacking:

...*when you tell someone ‘take your child for immunization’ she will simply tell you that ‘whenever she goes there [health facility] she does not find any drugs’...the woman loses morale instead and comes back*. (CHW, FGD).

... *[People] do not get what they are expecting when they reach [the health facility]. She leaves [home] sick knowing that she will get treated because she is a poor woman with no money and when she reaches there [health facility], the health work tells her... ‘I have written for you... go and buy drugs... there are no drugs here’*. (CHW, FGD).

Additionally, CHWs were discouraged when some community members had been asked for payment by health providers before receiving services:

*Health services are there but sometimes the common person who goes to access [antenatal care] may not afford to pay the charges. ... Because when she gets [to health facility], they will ask her for some money... It becomes a challenge for women when they go to deliver from health facility*... (CHW, FGD).

The practice of payments to health workers was seen by participants as a barrier to accessing reasonable services and negatively impacted CHW motivation where they had initiated a referral they deemed important. In some cases, CHWs described lowered morale since community members lost confidence in their recommendations, becoming hesitant to follow their advice.

*Availability of facility health providers:* Availability of facility health care providers to attend women and children was seen as a facilitator to sustainability of CHW networks. Some community members complained about continued facility shortages of medicine and tardiness/absence of health workers. CHWs who referred such patients reported feeling discouraged:

... *there are always few health workers who do not keep time...the facility is supposed open by 8:00am...you find them [health care providers] beginning work at 11:00am*. (CHW, FGD)

##### 2) CHW Program Factors

Factors related to CHW program structure and design were identified as key influencers of CHW sustainability. *CHW Selection and Training:* The CHW recruitment process was reported by community members as an enabler to CHW longevity and community service. Study participants expressed confidence that CHWs, selected from within and by the community itself, would remain. They expressed that CHW skills and knowledge gained through training continued to be shared with neighbors after the intervention ended.

*[The project] left when it had trained [CHWs]; even when [the project] ended, the CHWs continued working because they had experience through what they were taught. Because even if [the project] ended, the CHWs are in the village and they provide advice when they see a sick child or pregnant woman in the village*. (CHW, FGD)

*CHW Recognition and Incentives:* Participants explained that for CHW activities to be sustained, CHWs required community recognition. CHWs who were perceived as unimportant or illegitimate, or CHWs living in communities where the volunteer nature of their work was misunderstood, reported lower morale. Some respondents, especially CHWs, described that the small, non-financial incentives provided during the intervention, had facilitated CHW motivation at the time. Once incentives ceased post intervention, they reported declining motivation.

*Provision of incentives like t-shirts during the training motivated the CHWs*... (Health Unit Management Committee Chairperson, IDI).

*CHW Supervision:* Ongoing support to CHWs provided by facility-based supervisors was seen as critical for maintaining momentum of CHWs and community-based programming; supervisors who built trust were seen to facilitate long-term CHW network success.

CHW Refresher Training: Interval refresher training provided twice a year during the intervention was seen to promote CHW momentum and ongoing motivation. Decreased training opportunities post intervention were considered a barrier to sustainability.

*Refresher trainings were meant to refresh them [CHWs) and since the time they were trained...they have never got any other training. So, think of that. A person who is totally illiterate, does not know how to read and write, if you do not make a refresher training, he or she forgets everything. Even filling the register becomes a problem... they have totally forgotten because other organizations which come they do not train them, that's the most painful part of it*. (Health Assistant, IDI).

##### 3) Community attitudes and beliefs

For CHW networks and health outcomes to be maximized and sustained, respondents noted the importance of communities understanding CHW roles and responsibilities. Some community members were early adopters and engaged easily with CHWs; others only engaged when ‘incentivized’. Other community members continued to hold deeply rooted beliefs and attitudes that conflict with CHW messaging. To facilitate CHW program sustainability, there is need to engage community members at intervention start. This enables appreciation of the potential intervention benefits without compromising their beliefs and roles of CHWs in the intervention.

*Community Engagement:* Solid engagement of the broader community at intervention start was seen as a facilitator, supporting CHW community roles post-intervention; sensitization about potential benefit and roles of CHWs increased health promotion uptake both during and following the implementation phase.

*Sometimes, a programme can be introduced in a certain community but there is no awareness and there is no mobilisation. People do not know that the programme is going to help them, people do not know their responsibility in that programme, the role they are supposed to play and the role of implementers. For programmes [health related] to work, people should know their role, as the beneficiaries and the implementers know their role. So when people [community members] are missing that knowledge of course they cannot accept such a programme*. (Health worker, IDI).

Some community members were reported to be only interested in engaging with CHWs when provided with an incentive; for example, a bar of soap or a packet of salt. *People in our community thought we were being paid salary not working as volunteers...it became a challenge; you mobilize ten people only two will come if you are lucky. They want to be “[provided an incentive]”, when for us [we] have received nothing*... (CHW, FGD)

*Community Beliefs:* Persistent and traditional beliefs contradicting CHW health promotion messaging challenged long-term uptake and reducing potential for outcomes amongst certain populations especially after implementation since the CHWs messages lessen or stop with implementation)

*Talking about family planning is a challenge. Community members say that when one uses family planning pills for a long time, they affect their performance [sexual performance]. That is what people say and we are not technical persons to give the right information*. (Community Development Officer, IDI).

*Among the community members, the problem we find, they have what we call preconceived opinions...they think when they go to health facilities they will find young midwives delivering them...they have trust in the old women, traditional birth attendants* (Health Assistant, IDI).

##### 4) Stakeholder Engagement

Integration within existing government structures was described to be a key sustainability facilitator; the extent of alignment with programs and priorities and use of existing government structures was critical.

*Alignment with District Priorities and Programs:* District involvement enabled intervention sustainability. Respondents described enhanced long-term government support and prioritization for MNCH resulting from active district leadership and involvement during project implementation. Increased MNCH-allocated resources, CHW integration within district health programs, CHW supervisor support, and improved medical supply chain management all reportedly enabled continued CHW momentum.

*District health officers and other health leaders have helped in the way of integration of [MNCH] programs. For example, in case of other meetings they get involved in and they discuss about the same interventions like immunization. They are the ones who know and bring the challenges of health interventions to the technical planning committee meetings and others such that they are funded. So they lobby for these interventions*. (Community Development Officer, IDI)

*Local Government Involvement:* Participants linked continuing CHW effectiveness over time with the level of local government leader support. A CHW describes his/her CHW team experiencing resistance when not well-supported by elected community leaders:

... *Instead of moving together as a team in what we were doing to develop our area, some [local leaders] thought we are paid salary. Others thought we are taking away their responsibilities, so instead of [the project] growing stronger, they started opposing us. You know local leaders have a lot of influence, if he/she does not support you, you may not do much on the ground. For example, instead of them advising fellow men to go with their wives for antenatal, they are not there, they are not advising fellow men on having toilets at their homes, they are not there to advise men who refuse to build kitchens and renovate their homes. For us we advise; we are not law enforcers*. (CHW, FGD).

CHWs complained that where allowances were not available for government officials, some hesitated to participate in community-based health activities, jeopardizing project impact:

*Sometimes when you go to the [elected official] ...he will say “your community programs with no allowances waste our time” ...some [local leaders] will not be interested and will not help at all*. (CHW, FGD).

## Discussion

Five years post CHW intervention, key factors positively and negatively affect longevity of a district-wide CHW program in rural Uganda. Health system factors, CHW program factors, community attitudes and beliefs and stakeholder engagement were of critical importance in maintaining momentum of an initially effective community-based intervention. To the best of our knowledge, this is one of few studies in East Africa to assess CHW program sustainability factors in the medium term.

Within each theme, we identified components that affected long-term sustainability of CHW networks according to insights from those closest to these individuals who volunteer their time to serve the health needs of their communities. For all the themes, there were examples of successes and failures that suggest potential steps to mitigate the barriers even in a low resource setting. Some factors which were barriers to sustainability may have been mitigated at the implementation stage including gaps in engaging local leaders, unclear expectations and understanding of the volunteer nature of CHWs by community members. Other barriers demonstrate the importance of health system strengthening alongside community-based programming.

For example, as demand increases, facility-based services and health systems must support increased care-seeking and community demand through equipping health facilities with medical supplies and ensuring availability of health care providers otherwise CHWs can be demoralized. Conversely, projects should seek opportunities to promote those factors which enhance CHW sustainability, both during the life of a project and afterwards. Maximizing local leader support, promoting integration of CHWs and other community activities within existing health and political structures are some of the factors that can promote sustainability of CHWs. Provision of refresher training was a major motivator and sustainability factor, however, the short-term ‘output-driven’ format of most programs and grants does not easily provide opportunities for this.

Sustainability enablers identified in this study are consistent with the literature e.g. community ownership of the health interventions because this enables the local stakeholders to own the intervention having contributed to its implementation and the benefits involved^46^,^47^. Additionally, working within existing community systems enables sustainability of health interventions since the foundation of the intervention depends on resources generated in the community^48^,^49^. The spirit of volunteerism from the community members and existing infrastructure are further factors contributing towards sustainability of health interventions in the community^50^,^51^. On the other hand, weak health systems, lack of financial leadership and failure to delegate responsibilities to the local people negatively impacts sustainability of health interventions^52^,^53^. Financial support and integration of health interventions in the local government budgets was reported as one factor enabling sustainability of health interventions similar to reports from where and when^54^. In addition, training and empowerment of CHWs to carry on with the implemented interventions was mentioned as a major factor enabling sustainability of health interventions in the community. This was further supported by reports that refresher trainings of the community health workers further motivates them to continue their work in the community^51^.

Our findings on factors hindering sustainability of CHW programs are in line with what has been reported in previous studies. Lack of incentives to motivate CHWs and lack of supervision have been reported in previous research as major barriers to sustainability of community health interventions^55^,^56^. Lack of incentives demotivated CHWs especially when they had to move long distances to access households without transport fare similar to what was reported in our study^55^. While CHWs who are at the forefront of these interventions usually need supervision from facility health workers, facility health workers are not usually available at the health facilities. This is similar to what has been found in previous research^36^,^56^. It has been reported in some studies that facility health workers are not aware of what their supervisory role entails and this may hinder sustainability of health interventions due to the fact that the supervisory roles assigned may not be appropriate to what the CHWs need to function effectively^35^. Another barrier to sustainability of health interventions in the community as cited in our study is lack of support from the community members. This has been attributed to lack of sensitization bearing in mind that community members have their own beliefs and customs that contradict modern medications. Similar findings were reported in a study in Zaire^57^.

Community mobilization and sensitization at all stages of the implementation of the interventions plays a major role in the sustainability of the interventions. Whenever community members are not involved they oppose the interventions and they do not offer support to the CHWs contrary to the feeling of ownership whenever they are involved from the start^47^,^58^. Moreover whenever community members are involved from the start the sense of ownership propels them to support the interventions due to the fact that they appreciate the benefits especially when they view themselves as having contributed towards disease prevention in the community^49^. While failure to involve major stakeholders including local leaders and religious leaders was not a barrier to sustainability of health interventions in our study, it has been reported that involvement of these stakeholders during implementation fosters sustainability due to the fact these stakeholders get to influence the interventions so that they can be tailored to the needs of the community^49^. Previous research noted that involvement of church leaders has been instrumental in sustainability of health interventions in the community^48^.

## Conclusions

Global investments in CHW programming are enormous; understanding factors impacting sustainability is critical for programmers, funders and policy-makers. Our study identified important factors affecting CHW program sustainability which can and must be mitigated if CHWs are to be effective and sustainable. Early and meaningful engagement of community leaders, influencers and community members and strengthened linkages and accountability of health facilities to their communities and CHWs can and should be done to maximize the medium and long-term sustainability and thus impact the dedicated and important CHW cadre in rural Uganda and globally.

## Data Availability

The datasets collected and analyzed are presented in this manuscript.
